# Increased plasma levels of heparin-binding protein in patients with acute respiratory distress syndrome

**DOI:** 10.1186/cc12834

**Published:** 2013-07-24

**Authors:** Qionghua Lin, Jie Shen, Lihua Shen, Zhongwei Zhang, Fengming Fu

**Affiliations:** 1 Department of Anaesthesia, Critical Care and Pain Medicine, Fudan University Shanghai Cancer Center, Shanghai 200032, China; 2 Department of Oncology, Shanghai Medical College, Fudan University, Shanghai 200032, China; 3 Department of Anaesthesia, Critical Care and Pain Medicine, Fudan University Jinshan Hospital, 1508 Longhang Road, Shanghai 201508, China

**Keywords:** Acute lung injury, Acute respiratory distress syndrome, Cardiogenic pulmonary edema, Heparin-binding protein

## Abstract

**Introduction:**

Heparin-binding protein (HBP) is an antimicrobial protein stored in neutrophil granules and plays a role in endothelial permeability regulation. The aim was to assess the diagnostic and prognostic value of measuring HBP in patients with acute lung injury (ALI)/acute respiratory distress syndrome (ARDS).

**Methods:**

Plasma HBP was collected from 78 patients with ALI/ARDS, 28 patients with cardiogenic pulmonary edema (CPE) and 20 healthy volunteers at enrollment. Levels of HBP were measured by ELISA.

**Results:**

Patients with ALI/ARDS had significantly higher median levels of HBP compared with patients with CPE (17.15 (11.95 to 24.07) ng/ml vs. 9.50 (7.98 to 12.18) ng/ml, *P* <0.001) at enrollment. There was no significant difference between CPE patients and healthy subjects in terms of HBP value (*P* = 0.372). The HBP levels of nonsurvivors was significantly higher than that of survivors (23.90 (14.81 to 32.45) ng/ml vs. 16.01 (10.97 to 21.06) ng/ml, *P* = 0.012) and multivariate logistic regression showed HBP (odds ratio =1.52, *P* = 0.034) was the independent predictor for 30-day mortality in patients with ALI/ARDS.

**Conclusions:**

Plasma HBP levels of ALI/ARDS patients were significantly higher than that of CPE patients. HBP was a strong prognostic marker for short-term mortality in ALI/ARDS.

## Introduction

Acute lung injury (ALI)/acute respiratory distress syndrome (ARDS) is a common cause of life-threatening acute respiratory failure in ICUs worldwide. ALI/ARDS is characterized by systemic inflammation, disruption of endothelial and alveolar epithelial barriers, and an increase in microvascular permeability, resulting in pulmonary edema and respiratory failure. Diagnosis of ALI/ARDS therefore requires the exclusion of cardiogenic pulmonary edema, which is usually done on the basis of clinical judgment along with echocardiography and invasive hemodynamic monitoring.

There has been great interest in defining markers of prognosis or pathophysiology in ALI/ARDS. Heparin-binding protein (HBP), also known as azurocidin or CAP37, is an antimicrobial protein stored in neutrophil granules. HBP induces cytoskeletal rearrangement of endothelial cells, which leads to breakdown of cell barriers and an increase of the macromolecular efflux [1]. Convincing evidence has shown that HBP is a multifunctional protein. First, it has a broad spectrum of antimicrobial activity, mainly against Gram-negative bacteria [2]. Second, it has potent chemoattractant activity for monocytes, fibroblasts, T lymphocytes and, to some extent, neutrophils [3-5]. In addition, HBP has an exclusive role in mediating blood vessel wall permeability evoked by β_2_-integrin-mediated neutrophil activation [1]. Elevated plasma levels of HBP were found in patients with severe sepsis and septic shock [6-8]. In addition, studies have indicated that HBP may play an important role in regulating monocyte accumulation in the lung during acute inflammation [9]. HBP appears to be one of the primary effector molecules of transfusion-related acute lung injury [10]. Considering the effect of HBP on the capillary permeability and its release by activated neutrophils, we investigated the plasma levels of HBP and analyzed its value in patients with ALI/ARDS.

## Materials and methods

### Study population

The prospective, observational trial was undertaken from May 2011 to June 2012. A total of 106 consecutive patients with ALI/ARDS or cardiogenic pulmonary edema (CPE) admitted into the ICU of Fudan University Jinshan Hospital (Shanghai, China) were included. Exclusion criteria included age <18 years, primary abnormalities of coagulation, fibrinolytic therapy, immunosuppression due to medication or disease and patients on hemodialysis. Healthy volunteers (*n* = 20, free from pulmonary or cardiac disease) were defined as normal individuals. The study protocol was approved by the ethics committee of Fudan University Jinshan Hospital in accordance with the Declaration of Helsinki. Written informed consent was obtained from all participating people. The informed consent form was subscribed by the patient or their legally authorized representative when they were mechanically ventilated.

Two intensivists blinded to the results of HBP reviewed all other available clinical information integrated with progression of the disease and response to therapy, and then made the final diagnosis of ALI/ARDS or CPE. In case of disagreement, they reviewed the disputed cases together and reached a consensus. Patients whose diagnosis could not reach a consensus were excluded. Patients with ALI/ARDS met the following consensus definition: presence of a risk factor (such as sepsis, aspiration, shock and multiple transfusion), acute onset of bilateral infiltrates on chest radiography, severe hypoxemia with ratio of arterial oxygen partial pressure and inspiratory oxygen fraction (PO_2_/FiO_2_) <300 for ALI and <200 for ARDS [11]. Patients who had a mixed cause of pulmonary edema, with risk factors and criteria for ARDS/ALI except for a transient elevation in pulmonary artery occlusion pressure to 18 mmHg or higher, were included. We did not adopt the new Berlin definition of ARDS because our research was initiated from 2011 [12]. Patients with CPE were diagnosed by a combination of clinical signs (gallop, jugular venous distension, systolic hypertension), radiographic findings (cardiothoracic ratio >0.53 and vascular pedicle width >65 mm), electrocardiographic findings (new ST-segment and T-wave changes), laboratory findings (elevated troponin T >0.1 ng/ml), and hemodynamic findings (pulmonary artery occlusion pressure ≥18 mmHg, decreased ejection fraction <45%, presence of severe left-sided valvular heart disease (aortic or mitral stenosis or regurgitation)), and the response to appropriate therapy (preload/afterload reduction, treatment of ischemia or inotropic agents) [13].

### Data collection

Patients underwent an initial clinical assessment at enrollment. Invasive hemodynamic monitoring, echocardiogram, pulmonary function and computed tomography angiography were performed according to the treating physician. Insertion of a Swan–Ganz catheter was required when the diagnosis was uncertain. Forty-nine patients (62.8%) with ALI/ARDS and 15 patients (53.6%) with CPE were intubated and mechanically ventilated. The tidal volume and frequency were adjusted aiming at normocapnia and plateau pressure <35 cmH_2_O when volume-controlled ventilated. When pressure-controlled ventilated, the tidal volume was limited not to exceed 10 ml/kg. The positive end-expiratory value was set at 5 cmH_2_O or more when needed, guided by an arterial oxygen partial pressure >60 mmHg. All patients were enrolled within 10 hours of the diagnosis of ALI/ARDS or CPE. Comorbidities, ventilatory data, hemodynamic and laboratory findings were recorded at enrollment. The Acute Physiology and Chronic Health Evaluation (APACHE) II score [14], the Lung Injury Score (LIS) [15] and the Sequential Organ Failure Assessment score [16] were also calculated. For some analysis, the causes of ALI/ARDS were classified into an infected group (infection as the cause) and a non-infected group (all other causes, including hemorrhagic shock, aspiration, resuscitation and multiple transfusion). In addition, the degree of hypoxemia of ALI/ARDS was divided into mild (200 <PO_2_/FiO_2_ ≤300), moderate (100 <PO_2_/FiO_2_ ≤200) and severe (PO_2_/FiO_2_ ≤100). The infected group was classified into no sepsis (infection without systemic inflammatory response syndrome (SIRS)), severe sepsis and septic shock, which was defined according to the international criteria [17]. Patients were followed for the primary end point of 30-day mortality and the secondary end points of the number of ventilator-free days (VFD) and nonpulmonary organ failure-free days over a 30-day period after enrollment.

Blood samples for determination of HBP were collected at enrollment for all participants and were centrifuged within the next 1 hour. Plasma samples were frozen at −80°C for further analysis. Levels of HBP were measured using an ELISA according to the manufacturer’s instructions (Human azurocidin ELISA Kit; R&D, Minneapolis, MN, USA). The plates were read at a wavelength of 450 nm with an automatic ELISA reader and the assay did not cross-react with other related protein.

### Statistical analysis

Continuous variables are presented as mean ± standard deviation or median (interquartile range), and categorical variables as numbers and percentages. Two groups were compared with an unpaired Student’s *t* test or Mann–Whitney test for continuous variables and a chi-square test for categorical variables. If the difference among multiple groups was indicated significant by the Kruskal–Wallis test, then the Mann–Whitney test was used to further evaluate the difference between two groups. Correlations among continuous variables were assessed by Spearman rank analysis. Receiver operating characteristic curves were utilized to evaluate the accuracy of HBP to diagnose ALI/ARDS and the value of HBP to predict 30-day mortality. The optimal cutoff value was determined when the Youden index reached the maximum value. Logistic regression was assessed by univariate and multivariate analysis to identify independent predictors of outcome. Linear regression models were evaluated for the outcomes of VFD and nonpulmonary organ failure-free days by log-transforming corresponding variables to achieve a normal distribution. All probabilities were two tailed and *P* <0.05 was regarded as significant. Data were statistically analyzed with SPSS 16.0 software (SPSS Inc., Chicago, IL, USA).

## Results

### Patient characteristics

A total of 126 people, 78 patients with ALI/ARDS, 28 patients with CPE and 20 healthy volunteers, were enrolled in the study. Six patients with an initial disagreement of diagnosis were reviewed to reach a consensus, and two patients whose diagnosis could not reach a consensus were excluded. Causes of ALI/ARDS included pneumonia in 38 patients (48.7%), nonpulmonary sepsis in 20 patients (25.6%), hemorrhagic shock in eight patients (10.3%), aspiration in six patients (7.7%), resuscitation in four patients (5.1%) and multiple transfusions in two patients (2.6%). Causes of CPE included congestive heart failure in 12 patients (42.9%), myocardial infarction/ischemia in nine patients (32.1%) and acute volume overload in seven patients (25.0%).

The baseline characteristics of the study population are presented in Table 1 stratified according to the final diagnosis. Compared with patients with ALI/ARDS, patients with CPE were more likely to have a history of atrial fibrillation, lower Sequential Organ Failure Assessment score and higher PO_2_/FiO_2_. Echocardiographic and hemodynamic data indicated that patients with CPE had lower left ventricular ejection fraction and higher pulmonary artery occlusion pressure.

**Table 1:** Baseline characteristics of patients with ALI/ARDS or CPE

**Characteristic**	**ALI/ARDS (*n *** **= 78)**	**CPE (*n *** **= 28)**	***P *** **value**
Demographics			
Age (years)	63 (54 to 68)	66 (58 to 72)	0.231
Male	45 (57.7)	15 (53.6)	0.706
Body mass index (kg/m^2^)	23 (19 to 26)	23 (18 to 26)	0.297
Comorbidities			
Post operation	16 (20.5)	4 (14.3)	0.659
Atrial fibrillation	5 (6.4)	6 (21.4)	0.025*
Coronary artery disease	13 (16.7)	7 (25.0)	0.334
Chronic obstructive pulmonary disease	14 (17.9)	3 (10.7)	0.552
Hypertension	22 (28.2)	6 (21.4)	0.485
Diabetes	20 (25.6)	5 (17.9)	0.405
Chronic kidney disease	8 (10.3)	2 (7.1)	0.915
APACHE II score	18 (14 to 22)	15 (13 to 18)	0.067
SOFA score	8 (5 to 10)	4 (3 to 7)	0.037*
Ventilatory data			
Mechanical ventilation	49 (62.8)	15 (53.6)	0.391
Arterial pH	7.36 (7.31 to 7.42)	7.41 (7.37 to 7.48)	0.398
Arterial lactate	1.8 (1.1 to 2.3)	1.5 (1.2 to 1.9)	0.165
PO_2_/FiO_2_	166 (132 to 202)	224 (176 to 268)	0.033*
Echocardiography			
Patients examined	65 (83.3)	25 (89.3)	0.450
LVEF (%)	55 (51 to 61)	48 (41 to 53)	0.038*
Hemodynamics			
Patients examined	31 (39.7)	12 (42.9)	0.773
PAOP (mmHg)	14 (9 to 16)	23 (21 to 25)	0.042*
Cardiac index (l/min/m^2^)	3.01 (2.55 to 3.18)	2.85 (2.56 to 3.12)	0.671
SvO_2_ (%)	61 (53 to 66)	67 (60 to 73)	0.091
Laboratory findings			
White blood cell (10^9^/l)	12.1 (8.6 to 14.8)	9.3 (6.3 to 12.5)	0.315
Platelet (10^9^/l)	182 (137 to 255)	191 (155 to 257)	0.871
APTT (seconds)	30 (24 to 37)	31 (26 to 35)	0.672
Bilirubin (μmol/l)	11.7 (7.2 to 16.8)	9.1 (6.8 to 13.9)	0.774
Glucose (mmol/l)	6.7 (5.2 to 7.0)	6.5 (5.3 to 6.9)	0.781
Albumin (g/l)	28.2 (26.1 to 30.6)	30.2 (26.1 to 33.8)	0.317
Creatinine (μmol/l)	71 (55 to 88)	76 (55 to 91)	0.225
HBP (ng/ml)	17.15 (11.95 to 24.07)	9.50 (7.98 to 12.18)	<0.001*
Length of ICU stay (days)	9 (5 to 15)	7 (4 to 12)	0.221

Data presented as median (interquartile range) for continuous variables and number (%) for categorical variables. ALI, acute lung injury; APACHE, Acute Physiology and Chronic Health Evaluation; APTT, activated partial thromboplastin time; ARDS, acute respiratory distress syndrome; CPE, cardiogenic pulmonary edema; HBP, heparin-binding protein; LVEF, left ventricular ejection fraction; PAOP, pulmonary artery occlusion pressure; PO_2_/FiO_2_, ratio of arterial oxygen partial pressure and inspiratory oxygen fraction; SOFA, Sequential Organ Failure Assessment; SvO_2,_ oxygen saturation of venous blood. *P <0.05.

### Heparin-binding protein values

**Figure 1:**
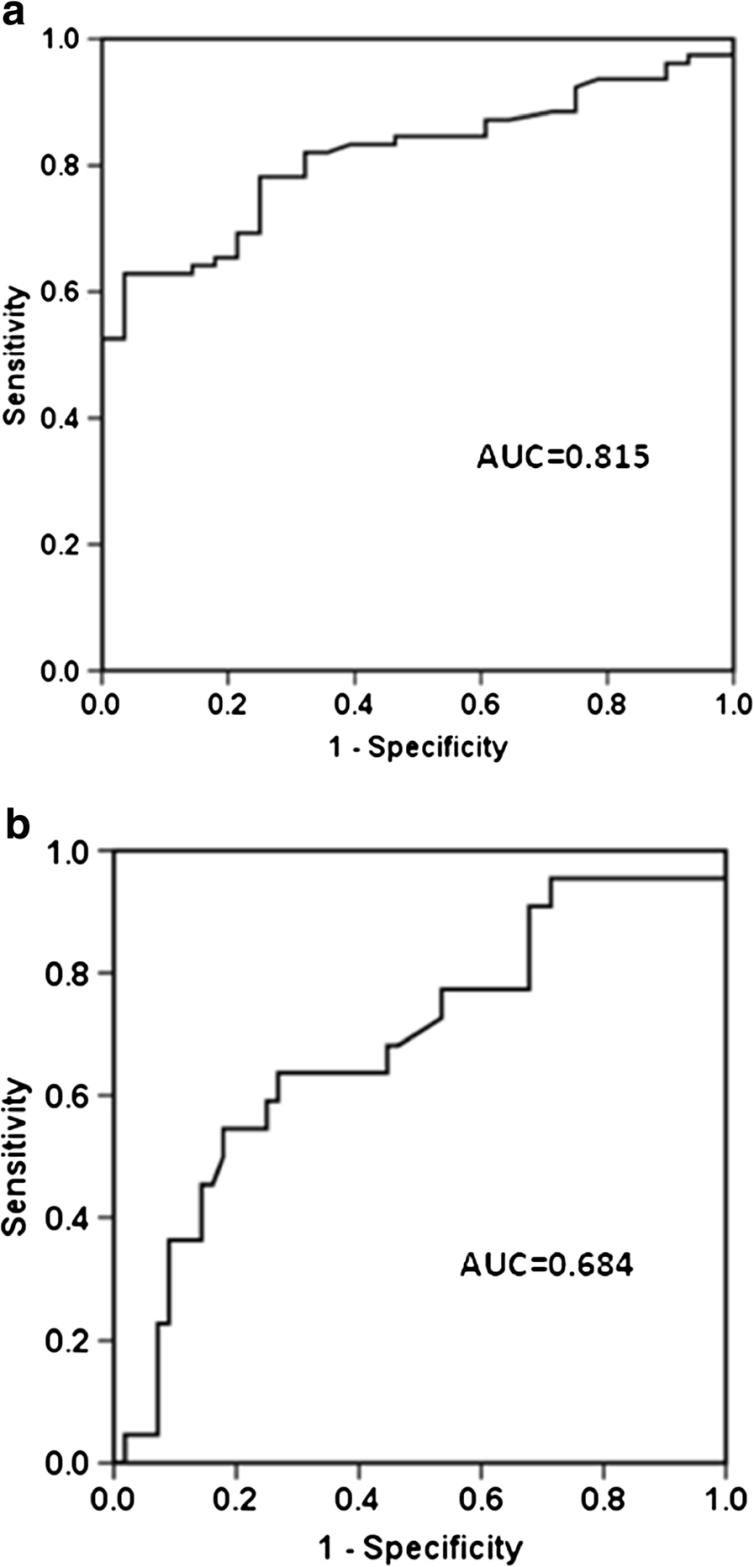
**Heparin-binding protein for disease diagnosis and mortality prediction.** Receiver operating characteristic curves for heparin-binding protein in **(a)** diagnosing acute lung injury (ALI)/acute respiratory distress syndrome (ARDS) from cardiogenic pulmonary edema and **(b)** predicting 30-day mortality in patients with ALI/ARDS. AUC, area under the curve.

Patients with ALI/ARDS had significantly higher median levels of HBP compared with patients with CPE (17.15 (11.95 to 24.07) ng/ml vs. 9.50 (7.98 to 12.18) ng/ml, *P* <0.001) and compared with healthy subjects (17.15 (11.95 to 24.07) ng/ml vs. 6.75 (4.33 to 10.65) ng/ml, *P* <0.001) at enrollment. There was no significant difference between CPE patients and healthy subjects in terms of HBP value (*P* = 0.372). The area under the receiver operating characteristic curve for HBP in relation to the final diagnosis of ALI/ARDS from CPE was 0.815 ± 0.040 (Figure 1a). At a cutoff point >11.55 ng/ml, HBP provided specificity of 78.2% and sensitivity of 75.0% for the diagnosis of ALI/ARDS.

Patients with ALI/ARDS were classified into an infected group of 58 patients (74.4%) and a non-infected group of 20 patients (25.6%). The median HBP levels at enrollment were 17.65 (11.66 to 25.23) ng/ml and 16.51 (12.03 to 24.06) ng/ml, respectively; the difference did not reach statistical significance (*P* = 0.806). Furthermore, the difference between the infected group with severe sepsis/septic shock and the non-infected group was not significant either (18.62 (12.08 to 28.10) ng/ml vs. 16.51 (12.03 to 24.06) ng/ml, *P* = 0.678).

**Figure 2:**
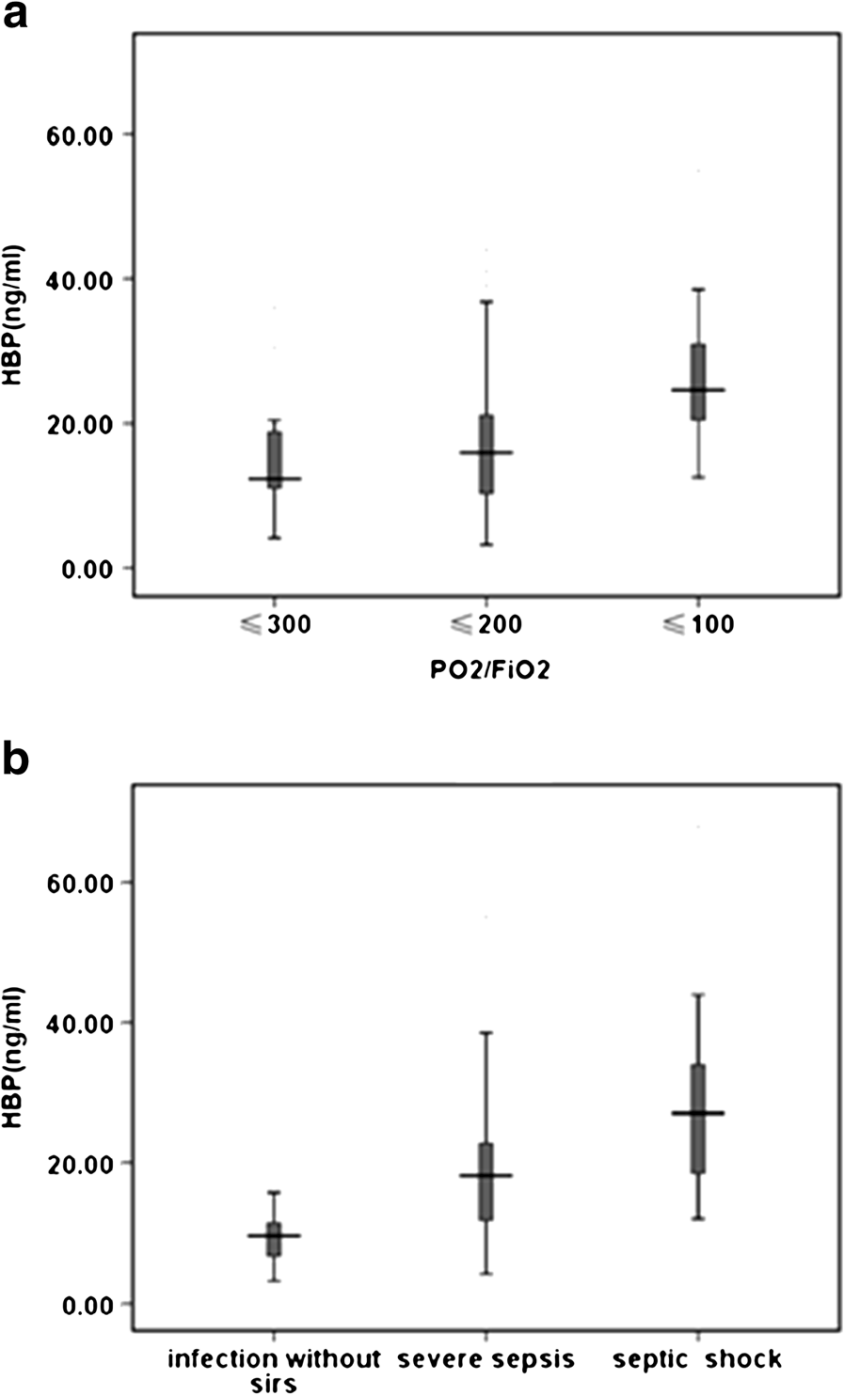
**Heparin-binding protein levels classified by arterial oxygen partial pressure/inspiratory oxygen fraction ratio and by infection.** Heparin-binding protein (HBP) levels in patients with acute lung injury/acute respiratory distress syndrome classified into **(a)** ratio of arterial oxygen partial pressure and inspiratory oxygen fraction (PO_2_/FiO_2_) ≤300, PO_2_/FiO_2_ ≤200, PO_2_/FiO_2_ ≤100 and **(b)** infection without systemic inflammatory response syndrome (SIRS), severe sepsis and septic shock.

ALI/ARDS patients were divided into mild (*n* = 20), moderate (*n* = 39) and severe (*n* = 19) groups according the degree of hypoxemia. There was no significant difference in HBP value between the mild and moderate groups (12.35 (11.07 to 19.15) ng/ml vs. 16.02 (9.82 to 21.23) ng/ml, *P* = 0.396). However, the HBP value of the moderate group was significantly lower than that of the severe group (16.02 (9.82 to 21.23) ng/ml vs. 24.60 (19.04 to 32.05) ng/ml, *P* = 0.013) (Figure 2a).

The infected group of ALI/ARDS patients were classified into no sepsis (infection without SIRS, *n* =7), severe sepsis (*n* =40) and septic shock (*n* =11). The HBP levels of patients with severe sepsis were significantly higher than those of patients with infection without SIRS (18.14 (11.79 to 22.95) ng/ml vs. 9.56 (6.34 to 12.92) ng/ml, *P* = 0.015). Nevertheless, the difference of HBP levels between patients with severe sepsis and septic shock was not significant (27.12 (16.03 to 36.01) ng/ml vs. 18.14 (11.79 to 22.95) ng/ml, *P* = 0.090) (Figure 2b). In addition, we found no significant difference in HBP levels of ALI/ARDS patients with septic and nonseptic shock (27.12 (16.03 to 36.01) ng/ml vs. 21.07 (11.98 to 31.54) ng/ml, *P* = 0.387).

In patients with ALI/ARDS, HBP levels were correlated with serum creatinine (*r* = 0.67, *P* <0.001), PO_2_/FiO_2_ (*r* = −0.58, *P* = 0.033), APACHE II score (*r* = 0.42, *P* = 0.022), and lactate (*r* = 0.40, *P* = 0.048) at enrollment.

### Outcome

**Figure 3:**
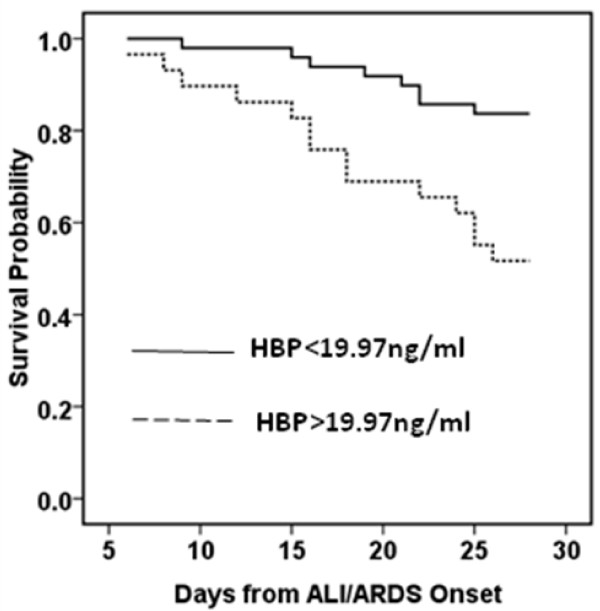
**Survival probability by heparin-binding protein value.** Kaplan–Meier survival probability by heparin-binding protein (HBP) value above or below the cutoff point of 19.97 ng/ml. ALI, acute lung injury; ARDS, acute respiratory distress syndrome.

The mortality was 28.2% in patients with ALI/ARDS. Compared with survivors, nonsurvivors were significantly older, had higher APACHE II score, higher Sequential Organ Failure Assessment score and lower PO_2_/FiO_2_ (Table 2). The HBP levels of nonsurvivors were significantly higher than those of survivors (23.90 (14.81 to 32.45) ng/ml vs. 16.01 (10.97 to 21.06) ng/ml, *P* = 0.012) at enrollment. The receiver operating characteristic curve was drawn to evaluate the value of HBP to predict 30-day mortality and the area under the curve was calculated as 0.684 ± 0.069 (Figure 1b). The optimal cutoff value for predicting death was >19.97ng/ml, which gave specificity of 73.2% and sensitivity of 63.6%. A Kaplan–Meier curve was drawn according to the value of 19.97 ng/ml for HBP as a cutoff point to describe death over 30 days of follow-up (Figure 3). There was a significant difference in the occurrence of death (*P* = 0.002).

**Table 2:** Baseline characteristics of patients with ALI/ARDS according to survival

**Characteristic**	**Nonsurvivors (*n *** **= 22)**	**Survivors (*n *** **= 56)**	***P *** **value**
Demographics			
Age (years)	68 (63 to 72)	60 (51 to 64)	0.041*
Male	13 (59.1)	32 (57.1)	0.875
Body mass index (kg/m^2^)	23 (20 to 24)	24 (20 to 26)	0.510
Main cause of ALI/ARDS			
Pneumonia	9 (40.9)	29 (51.8)	0.387
Nonpulmonary sepsis	7 (31.8)	13 (23.2)	0.434
Hemorrhagic shock	3 (13.6)	5 (8.9)	0.840
Aspiration	2 (9.1)	4 (7.1)	0856
APACHE II score	21 (18 to 23)	15 (12 to 18)	0.036*
SOFA score	8 (6 to 10)	5 (3 to 6)	0.041*
LIS score	2.4 (1.7 to 3.1)	1.8 (1.2 to 2.6)	0.087
Ventilatory data			
Mechanical ventilation	17 (77.3)	32 (57.1)	0.098
Arterial pH	7.29 (7.21 to 7.36)	7.39 (7.32 to 7.45)	0.128
Arterial lactate	2.0 (1.7 to 2.3)	1.6 (1.3 to 1.8)	0.219
PO_2_/FiO_2_	142 (120 to 171)	189 (158 to 217)	0.045*
Echocardiography			
Patients examined	17 (77.3)	48 (85.7)	0.368
LVEF (%)	53 (49 to 55)	56 (50 to 62)	0.271
Hemodynamics			
Patients examined	12 (54.5)	19 (33.9)	0.094
PAOP (mmHg)	14 (10 to 17)	12 (7 to 15)	0.652
Cardiac index (l/min/m^2^)	2.88 (2.41 to 3.05)	3.15 (2.66 to 3.31)	0.872
SvO_2_ (%)	58 (52 to 63)	63 (57 to 68)	0.529
Laboratory findings			
White blood cells (10^9^/l)	14.8 (11.6 to 16.2)	11.2 (9.7 to 14.6)	0.198
Platelets (10^9^/l)	165 (138 to 198)	211 (159 to 258)	0.265
APTT (seconds)	34 (28 to 37)	29 (25 to 33)	0.436
Bilirubin (μmol/l)	13.1 (10.1 to 15.1)	9.6 (7.2 to 12.8)	0.196
Glucose (mmol/l)	6.9 (5.6 to 7.4)	6.5 (5.4 to 7.0)	0.398
Albumin (g/l)	27.1 (25.8 to 29.6)	31.5 (28.1 to 33.4)	0.233
Creatinine (μmol/l)	81 (72 to 96)	72 (56 to 88)	0.378
HBP (ng/ml)	23.90 (14.81 to 32.45)	16.01 (10.97 to 21.06)	0.012*

Data presented as median (interquartile range) for continuous variables and number (%) for categorical variables. ALI, acute lung injury; APACHE, Acute Physiology and Chronic Health Evaluation; APTT, activated partial thromboplastin time; ARDS, acute respiratory distress syndrome; HBP, heparin-binding protein; LIS, Lung Injury Score; LVEF, left ventricular ejection fraction; PAOP, pulmonary artery occlusion pressure; PO_2_/FiO_2_, ratio of arterial oxygen partial pressure and inspiratory oxygen fraction; SOFA, Sequential Organ Failure Assessment; SvO_2,_ oxygen saturation of venous blood. *P <0.05.

Univariate logistic regression analysis showed that age, APACHE II score, LIS, PO_2_/FiO_2_ and plasma HBP levels at enrollment were the common predictors of 30-day mortality in patients with ALI/ARDS. Multivariate logistic regression analysis showed LIS (odds ratio = 1.61, *P* = 0.037), HBP (odds ratio = 1.52, *P* = 0.034) and PO_2_/FiO_2_ (odds ratio = 0.68, *P* = 0.041) remained the independent predictor for mortality after adjustment for risk factors (age, APACHE II score) (Table 3).

**Table 3:** Logistic regression analysis of mortality prediction for patients with ALI/ARDS

	**Univariate analysis**		**Multivariate analysis**	
	**OR (95% CI)**	***P *** **value**	**OR (95% CI)**	***P *** **value**
Age (per year)	1.09 (1.03 to 1.18)	0.042		
APACHE II score (per point)	1.17 (1.09 to 1.35)	0.033		
LIS (per point)	1.72 (1.18 to 3.12)	0.029	1.61 (1.18 to 2.77)	0.037
PO_2_/FiO_2_^a^ (per one-log)	0.61 (0.48 to 0.82)	0.031	0.68 (0.53 to 0.87)	0.041
HBP^a^ (per one-log)	1.65 (1.18 to 3.23)	0.027	1.52 (1.12 to 2.85)	0.034

ALI, acute lung injury; APACHE, Acute Physiology and Chronic Health Evaluation; ARDS, acute respiratory distress syndrome; CI, confidence interval; HBP, heparin-binding protein; LIS, Lung Injury Score; OR, odds ratio; PO_2_/FiO_2_, ratio of arterial oxygen partial pressure and inspiratory oxygen fraction. ^a^Log-transformed.

In patients with ALI/ARDS, higher baseline plasma HBP levels were strongly associated with fewer VFD (*P* = 0.031) in 49 patients who received mechanical ventilation in analysis controlling for APACHE II score, LIS score, PO_2/_FiO_2_ and creatinine value. The HBP value, however, was not associated with nonpulmonary organ failure-free days in 52 patients who had at least one nonpulmonary organ failure in ALI/ARDS patients (Table 4).

**Table 4:** Linear regression analysis of outcomes for ventilator-free days/nonpulmonary organ failure-free days in patients with ALI/ARDS

	**Ventilator-free days**		**Nonpulmonary organ failure-free days**	
	**Standardized coefficient (β)**	***P *** **value**	**Standardized coefficient (β)**	***P *** **value**
APACHE II score			−0.566	0.022
LIS	−0.428	0.027		
PO_2_/FiO_2_	0.575	0.018		
Creatinine^a^			−0.196	0.046
HBP^a^	−0.297	0.031		

ALI, acute lung injury; APACHE, Acute Physiology and Chronic Health Evaluation; ARDS, acute respiratory distress syndrome; HBP, heparin-binding protein; LIS, Lung Injury Score; PO_2_/FiO_2_, ratio of arterial oxygen partial pressure and inspiratory oxygen fraction. ^a^Log-transformed to achieve a normal distribution.

## Discussion

HBP is a multifunctional protein with diverse and important activities including bactericidal capacity, anti-apoptotic properties and permeability-increasing activity. In our study, we found that patients with ALI/ARDS had significantly higher median levels of HBP compared with patients with CPE. Furthermore, HBP levels of nonsurvivors were significantly higher than those of survivors. Multivariate logistic regression analysis showed HBP at enrollment was the independent predictor for 30-day mortality.

HBP can be characterized as a proinflammatory protein that is rapidly released from emigrating neutrophil. HBP is chemotactic for monocytes, fibroblasts and T cells; moreover, HBP increases lipopolysaccharide-induced monocyte production of TNFα, IL-1 and IL-6 [18]. On the other side, HBP plays a role in endothelial permeability regulation; fibroblast and endothelia monolayers treated with HBP exhibited reversible disruption of barrier function by inducing cell contraction and the formation of large gaps between cells [19]. Linder and colleagues found that the HBP plasma concentration was already elevated in 29 of 32 severe septic patients before signs of circulatory failure appeared [6]. The association between high HBP levels and the development of sepsis with circulatory symptoms supports the role of HBP as a mediator of capillary leakage [20]. ALI/ARDS is a systemic disorder with endothelial activation and injury in both the systemic and pulmonary circulation. CPE is defined as pulmonary edema due to increased capillary hydrostatic pressure secondary to elevated pulmonary venous pressure [21]. The role of HBP in altering the vascular permeability was evoked by chemoattractant-induced polymorphonuclear leukocyte activation [1], which may account for the reason why the HBP levels did not increase in patients with CPE. HBP has been detected in lipopolysaccharide-treated endothelial cells (including human lung microvessel) as early as 2 hours and maximum staining was obtained between 4 and 6 hours [22]. In addition, HBP was demonstrated to augment leukocyte adhesion to lung microvessel endothelial monolayers and induce significant monocyte migration into lungs [9,23]. The expression of HBP in endothelial and epithelial cells in response to inflammatory mediators and its ability to alter permeability of endothelial cell was probably the interpretation of the high plasma HBP concentration in patients with ALI/ARDS in our research.

Observations have indicated that plasma HBP levels were significantly higher in patients with severe sepsis or septic shock compared with nonseptic patients [7]. A plasma HBP level >15 ng/ml was a better indicator of severe sepsis (with or without septic shock) than any other biomarker investigated (white blood cells, IL-6, procalcitonin and C-reactive protein) [6]. In the present study, HBP levels of patients with severe sepsis were significantly higher than patients with infection without SIRS. In addition, the difference in HBP levels between infected and non-infected patients with ALI/ARDS was not significant, and neither was the difference in patients with septic and nonseptic shock. Our results are consistent with previous research demonstrating that HBP levels were notably enhanced in patients with shock regardless of whether they suffered from sepsis or not [24]. This is supported by the viewpoint that not only bacterial products [25] but also nonbacterial cross-linking of β_2_-integrins on the surface of neutrophils is sufficient to trigger HBP secretion [1]. Serious events such as cardiac arrest or massive blood transfusions can also lead to neutrophil activation and the subsequent release of HBP [7], indicating that HBP is not entirely specific for infections.

There is controversy about the prognostic value of HBP: Linder and colleagues found that the 28-day case-fatality rate was increased fourfold among sepsis patients with initial HBP >15 ng/ml [7]. Nevertheless, another study showed that HBP levels did not correlate with survival in nonseptic and septic shock patients [24]. HBP has been shown to increase the survival of cultured monocytes and protect them from oxidative stress [4,26]. HBP-treated endothelial cells also demonstrated enhanced survival in apoptosis assays, suggesting that HBP may play a protective role during an inflammatory response [27]. However, the HBP expression may lead to the exacerbation or augmentation of the host immune response and progression of disease [28,29]. Furthermore, HBP was proved to shorten the prolonged clotting times of the activated partial thromboplastin time and the thrombin clotting time induced by a high concentration of unfractionated heparin [30], which may worsen the hypercoagulability with widespread microvascular thrombus formation in ALI/ARDS. In our study, the HBP value of the severe hypoxemia group was significantly higher than that of the moderate group and higher baseline plasma HBP levels were strongly associated with fewer VFD. HBP at enrollment was the independent predictor for 30-day mortality in our research, indicating HBP may be a good target of drugs for therapeutic intervention. Previous studies have revealed that both heparin and anti-HBP antibodies could block β_2_-integrin receptor on neutrophils, which is responsible for HBP release [1]. These treatments not only inhibit adhesion and activation of leukocyte but also attenuate neutrophil-evoked increase in endothelial permeability. Heparin has been confirmed to alleviate inflammatory reaction in ALI in rabbits by inhibiting overexpression of TNFα [31]. Moreover, nebulized heparin can significantly reduce coagulation activation in the lungs of critically ill patients with ALI [32]. A peptide (Gly-Pro-Arg-Pro) that interferes with the interaction between fibrinogen and β_2_-integrin could prevent severe lung lesions that developed in mice after streptococcal M1 protein injection [8], raising the question of whether this peptide could improve the survival of ALI/ARDS patients as an effect of blocking HBP and subsequent reduction in vascular leakage.

There are several limitations to our study. First, the study sample was small in size and composition, thus restricting generalizability. Second, the absence of a single-objective gold standard for diagnosis of ARDS is a challenge inherent to all studies of diagnostic testing for this syndrome. Finally, we did not test the concentration of HBP in alveolar fluid and lack an extended comparison with other biomarker of ALI/ARDS.

## Conclusion

Our findings suggest that HBP is a biomarker that could distinguish ALI/ARDS from CPE. Furthermore, HBP was a strong predictor for short-term mortality in ALI/ARDS. Whether HBP levels can be used to guide clinical management or shed further light on therapy of ALI/ARDS remains to be investigated.

## Key messages

• Patients with ALI/ARDS had significantly higher median levels of HBP compared with patients with CPE.

• HBP levels of nonsurvivors were significantly higher than survivors.

• HBP was the independent predictor for 30-day mortality in patients with ALI/ARDS.

## Abbreviations

ALI: Acute lung injury; APACHE: Acute Physiology and Chronic Health Evaluation; ARDS: Acute respiratory distress syndrome; CPE: Cardiogenic pulmonary edema; ELISA: Enzyme-linked immunosorbent assay; HBP: Heparin-binding protein; IL: Interleukin; LIS: Lung Injury Score; PO2/FiO2: Ratio of arterial oxygen partial pressure and inspiratory oxygen fraction; SIRS: Systemic inflammatory response syndrome; TNF: Tumor necrosis factor; VFD: Ventilator-free days.

## Competing interests

The authors declare that they have no competing interests.

## Authors’ contributions

QL collected the data, performed the statistical analysis and drafted the manuscript. LS and ZZ performed the experimental procedures and carried out the immunoassays. FF and JS participated in the study design and helped to draft the manuscript. All authors read and approved the final manuscript.

## References

[B1] Gautam N Olofsson AM Herwald H Iversen LF Lundgren-Akerlund E Hedqvist P Arfors KE Flodgaard H Lindbom L Heparin-binding protein (HBP/CAP37): a missing link in neutrophil-evoked alteration of vascular permeability Nat Med 2001 17 1123 1127 10.1038/nm1001-1123 11590435

[B2] Shafer WM Martin LE Spitznagel JK Late intraphagosomal hydrogen ion concentration favors the in vitro antimicrobial capacity of a 37-kilodalton cationic granule protein of human neutrophil granulocytes Infect Immun 1986 17 651 655 3527987 10.1128/iai.53.3.651-655.1986 PMC260843

[B3] Chertov O Ueda H Xu LL Tani K Murphy WJ Wang JM Howard OM Sayers TJ Oppenheim JJ Identification of human neutrophil-derived cathepsin G and azurocidin/CAP37 as chemo-attractants for mononuclear cells and neutrophils J Exp Med 1997 17 739 747 10.1084/jem.186.5.739 9271589 PMC2199011

[B4] Pereira HA Shafer WM Pohl J Martin LE Spitznagel JK CAP37, a human neutrophil-derived chemotactic factor with monocyte specific activity J Clin Invest 1990 17 1468 1476 10.1172/JCI114593 2332502 PMC296594

[B5] Chertov O Michiel DF Xu L Wang JM Tani K Murphy WJ Longo DL Taub DD Oppenheim JJ Identification of defensin-1, defensin-2 and CAP37/azurocidin as T-cell chemoattractant proteins released from interleukin-8 stimulated neutrophils J Biol Chem 1996 17 2935 2940 10.1074/jbc.271.6.2935 8621683

[B6] Linder A Christensson B Herwald H Björck L Akesson P Heparin-binding protein: an early marker of circulatory failure in sepsis Clin Infect Dis 2009 17 1044 1050 10.1086/605563 19725785

[B7] Linder A Akesson P Treutiger CJ Inghammar M Linnér A Sundén-Cullberg J Elevated plasma levels of heparin-binding protein in intensive care unit patients with severe sepsis and septic shock Crit Care 2012 17 R90 10.1186/cc11353 22613179 PMC3580636

[B8] Herwald H Cramer H Morgelin M Russell W Sollenberg U Norrby-Teglund A Flodgaard H Lindbom L Björck L M protein, a classical bacterial virulence determinant, forms complexes with fibrinogen that induce vascular leakage Cell 2004 17 367 379 10.1016/S0092-8674(04)00057-1 15016372

[B9] Doherty DE Nakano J Nakano K Neutrophil-derived heparin-binding protein – a monocyte-specific chemoatractant that induces monocyte migration into rabbit lungs in vivo Chest 1999 17 34S 35S 10424582 10.1378/chest.116.suppl_1.34s-a

[B10] Yasui K Furuta RA Matsuyama N Fukumori Y Kimura T Tani Y Shibata H Hirayama F Possible involvement of heparin-binding protein in transfusion related acute lung injury Transfusion 2008 17 978 987 18346022 10.1111/j.1537-2995.2007.01632.x

[B11] Bernard GR Artigas A Brigham KL Carlet J Falke K Hudson L Lamy M Legall JR Morris A Spragg R The American–European Consensus Conference on ARDS: definitions, mechanisims, relevant outcomes, and clinical trial coordination Am J Respir Crit Care Med 1994 17 818 824 10.1164/ajrccm.149.3.7509706 7509706

[B12] The ARDS Definition Task Force Acute respiratory distress syndrome: the Berlin definition JAMA 2012 17 2526 2533 22797452 10.1001/jama.2012.5669

[B13] Gropper MA Wiener–Kronish JP Hashimoto S Acute cardiogenic pulmonary edema Clin Chest Med 1994 17 501 515 7982344

[B14] Knaus WA Draper EA Wagner DP APACHE II: a severity of disease classification system Crit Care Med 1985 17 818 829 10.1097/00003246-198510000-00009 3928249

[B15] Murray JF Matthay MA Luce JM Flick MR An expanded definition of the adult respiratory distress syndrome Am Rev Respir Dis 1988 17 720 723 10.1164/ajrccm/138.3.720 3202424

[B16] Vincent JL Moreno R Takala J Willatts S De Mendonça A Bruining H Reinhart CK Suter PM Thijs LG The SOFA (Sepsis-related Organ Failure Assessment) score to describe organ dysfunction/failure Intensive Care Med 1996 17 707 710 10.1007/BF01709751 8844239

[B17] American College of Chest Physicians/Society of Critical Care Medicine Consensus Conference: definitions for sepsis and organ failure and guidelines for the use of innovative therapies in sepsis Crit Care Med 1992 17 864 874 10.1097/00003246-199206000-00025 1597042

[B18] Heinzelmann M Flodgaard H Miller FN Heparin-binding protein (CAP37) is internalized in monocytes and increases LPS-induced monocyte activation J Immunol 1998 17 5530 5536 9605157

[B19] Ostergaard E Flodgaard H A neutrophil-derived proteolytic inactive elastase homologue (hHBP) mediates reversible contraction of fibroblasts and endothelial cell monolayers and stimulates monocyte survival and thrombospond in secretion J Leukoc Biol 1992 17 316 323 1564396 10.1002/jlb.51.4.316

[B20] Linder A Soehnlein O Akesson P Roles of heparin-binding protein in bacterial infections J Innate Immun 2010 17 431 438 10.1159/000314853 20505311

[B21] Cardiogenic Pulmonary Edema http://emedicine.medscape.com/article/157452-overview

[B22] Lee TD Gonzalez ML Kumar P Chary-Reddy S Grammas P Pereira HA CAP37, a novel inflammatory mediator: its expression in endothelial cells and localization to atherosclerotic lesions Am J Pathol 2002 17 841 848 10.1016/S0002-9440(10)64907-3 11891183 PMC1867172

[B23] Lee TD Gonzalez ML Kumar P Grammas P Pereira HA CAP37, a neutrophil-derived inflammatory mediator, augments leukocyte adhesion to endothelial monolayers Microvasc Res 2003 17 38 48 10.1016/S0026-2862(03)00010-4 12826073

[B24] Chew MS Linder A Santen S Ersson A Herwald H Thorlacius H Increased plasma levels of heparin-binding protein in patients with shock: a prospective, cohort study Inflamm Res 2012 17 375 379 10.1007/s00011-011-0422-6 22207392

[B25] Påhlman LI Mörgelin M Eckert J Johansson L Russell W Riesbeck K Soehnlein O Lindbom L Norrby-Teglund A Schumann RR Björck L Herwald H Streptococcal M protein: a multipotent and powerful inducer of inflammation J Immunol 2006 17 1221 1228 16818781 10.4049/jimmunol.177.2.1221

[B26] Shrotri MS Kuhn JF Peyton JC Flodgaard HJ Klein JB Cheadle WG Heparin binding protein decreases apoptosis in human and murine neutrophils J Surg Res 2000 17 53 59 10.1006/jsre.1999.5803 10720453

[B27] Edens HA Parkos CA Neutrophil transendothelial migration and alteration in vascular permeability: focus on neutrophil-derived azurocidin Curr Opin Hematol 2003 17 25 30 10.1097/00062752-200301000-00005 12483108

[B28] Tapper H Karlsson A Mörgelin M Flodgaard H Herwald H Secretion of heparin-binding protein from human neutrophils is determined by its localization in azurophilic granules and secretory vesicles Blood 2002 17 1785 1793 10.1182/blood.V99.5.1785 11861296

[B29] Pereira HA Kumar P Grammas P Expression of CAP37, a novel inflammatory mediator, in Alzheimer’s disease Neurobiol Aging 1996 17 753 759 8892348

[B30] Kaiser P Harenberg J Walenga JM Huhle G Giese C Prechel M Hoppensteadt D Fareed J Effects of a heparin-binding protein on blood coagulation and platelet function Semin Thromb Hemost 2001 17 495 502 10.1055/s-2001-17960 11668419

[B31] Wang M He J Mei B Ma X Huo Z Therapeutic effects and anti inflammatory mechanisms of heparin on acute lung injury in rabbits Acad Emerg Med 2008 17 656 663 10.1111/j.1553-2712.2008.00146.x 19086324

[B32] Dixon B Santamaria JD Campbell DJ A phase 1 trial of nebulized heparin in acute lung injury Crit Care 2008 17 R6 10.1186/cc6770 18460218 PMC2481447

